# Effect of Acute Caffeine Intake on Fat Oxidation Rate during Fed-State Exercise: A Systematic Review and Meta-Analysis

**DOI:** 10.3390/nu16020207

**Published:** 2024-01-08

**Authors:** Javier Fernández-Sánchez, Daniel Trujillo-Colmena, Adrián Rodríguez-Castaño, Ana Myriam Lavín-Pérez, Juan Del Coso, Arturo Casado, Daniel Collado-Mateo

**Affiliations:** 1 Sport Sciences Research Centre, Rey Juan Carlos University, 28943 Madrid, Spain; javier.fernandezsa@urjc.es (J.F.-S.); daniel.trujillo@urjc.es (D.T.-C.); adrian.rodriguez@urjc.es (A.R.-C.); arturo.casado@urjc.es (A.C.); daniel.collado@urjc.es (D.C.-M.); 2 GO fitLAB, Ingesport, 28003 Madrid, Spain

**Keywords:** stimulant, supplementation, fat metabolism, aerobic exercise, breakfast

## Abstract

Pre-exercise intake of caffeine (from ~3 to 9 mg/kg) has been demonstrated as an effective supplementation strategy to increase fat oxidation during fasted exercise. However, a pre-exercise meal can alter the potential effect of caffeine on fat oxidation during exercise as caffeine modifies postprandial glycaemic and insulinemic responses. Hypothetically, the effect of caffeine on fat oxidation may be reduced or even withdrawn during fed-state exercise. The present systematic review aimed to meta-analyse investigations on the effect of acute caffeine intake on the rate of fat oxidation during submaximal aerobic exercise performed in the fed state (last meal < 5 h before exercise). A total of 18 crossover trials with randomised and placebo-controlled protocols and published between 1982 and 2021 were included, with a total of 228 participants (185 males and 43 females). Data were extracted to compare rates of fat oxidation during exercise with placebo and caffeine at the same exercise intensity, which reported 20 placebo–caffeine pairwise comparisons. A meta-analysis of the studies was performed, using the standardised mean difference (SMD) estimated from Hedges’ *g*, with 95% confidence intervals (CI). In comparison with the placebo, caffeine increased the rate of fat oxidation during fed-state exercise (number of comparisons (n) = 20; *p* = 0.020, SMD = 0.65, 95% CI = 0.20 to 1.20). Only studies with a dose < 6 mg/kg of caffeine (n = 13) increased the rate of fat oxidation during fed-state exercise (*p* = 0.004, SMD = 0.86, 95% CI = 0.27 to 1.45), while no such effect was observed in studies with doses ≥6 mg/kg (n = 7; *p* = 0.97, SMD = −0.03, 95% CI = −1.40 to 1.35). The effect of caffeine on fat oxidation during fed-state exercise was observed in active untrained individuals (n = 13; *p* < 0.001, SMD = 0.84, 95% CI = 0.39 to 1.30) but not in aerobically trained participants (n = 7; *p* = 0.27, SMD = 0.50, 95% CI = −0.39 to 1.39). Likewise, the effect of caffeine on fat oxidation was observed in caffeine-naïve participants (n = 9; *p* < 0.001, SMD = 0.82, 95% CI = 0.45 to 1.19) but not in caffeine consumers (n = 3; *p* = 0.54, SMD = 0.57, 95% CI = −1.23 to 2.37). In conclusion, acute caffeine intake in combination with a meal ingested within 5 h before the onset of exercise increased the rate of fat oxidation during submaximal aerobic exercise. The magnitude of the effect of caffeine on fat oxidation during fed-state exercise may be modulated by the dose of caffeine administered (higher with <6 mg/kg than with ≥6 mg/kg), participants’ aerobic fitness level (higher in active than in aerobically trained individuals), and habituation to caffeine (higher in caffeine-naïve than in caffeine consumers).

## 1. Introduction

Caffeine (1,3,7-trimethylxanthine) is a widely consumed psychoactive substance found in various food items, including chocolate and beverages such as coffee, tea, soft drinks, and energy drinks [1,2]. Caffeine is extensively used due to its properties to enhance the capacity to work and positively influence factors contributing to mood state such as concentration, vigour, and vitality perception. The benefits of caffeine have been attributed to its binding to adenosine receptors A_1_ and A_2a_ [3]. Upon this theory, caffeine blocks the “fatiguing” action of adenosine, a purine that generally inhibits neuronal activity [4]. This adenosine-blocking action of caffeine appears to contribute to the stimulatory effects of this substance on cognitive and physical performance [5]. Additionally, caffeine can induce other physiological actions in peripheral tissues facilitating that caffeine directly potentiates skeletal muscle force [6], among other effects.

Caffeine has been utilised as an ergogenic aid to enhance athletic performance in both anaerobic and aerobic exercise. Studies have demonstrated improvements in strength, power, speed, and jumping ability [5,7]. Furthermore, caffeine has been shown to improve cardiorespiratory endurance capacity during aerobic exercise [8]. In team sports, caffeine may also lead to performance improvements [9]. Additionally, the ergogenic effect of caffeine on cognitive function, attention, and vigilance has also been demonstrated [10]. Recommended doses to obtain such ergogenic benefits range from 3 to 9 mg/kg of body mass and its effect is observed in both trained and untrained individuals [5]. To obtain the ergogenic effect of oral administration, caffeine should be consumed approximately 60 min before the main activity [11]. 

Beyond ergogenicity, caffeine can also induce other physiological changes during exercise, particularly when exercise intensity is low to moderate. During submaximal aerobic exercise, caffeine can also enhance the use of fat as fuel for the contracting muscle to the detriment of the use of carbohydrates for the production of energy [12,13]. The mechanism by which caffeine may increase fat oxidation is through the activation of the sympathetic nervous system, which increases the release of catecholamines and a concomitant enhanced release of free fatty acids (FFA) and lipolysis [14]. A recent meta-analysis examined the effect of caffeine on the rate of fat oxidation in interventions of fixed exercise intensity in a fasted state (>5 h since the last meal), which reported a positive effect of caffeine on fat oxidation rates during fasted exercise [15]. Another meta-analysis has been recently published claiming that caffeine significantly improves fat metabolism at rest and during exercise [16]. In this latter meta-analysis [16], the effect of combining caffeine with fasting periods shorter and longer than 3 h before exercise was analysed and the report confirmed that caffeine enhances fat metabolism, independently of the fasting period used before exercise. However, this meta-analysis [16] included studies with very different natures, with a mix of studies carried out at rest and during exercise and with very diverse methods to assess fat metabolism (gas exchange, blood metrics of triglycerides, FFA, and glycerol, among others). Additionally, an increase in fat metabolism induced by caffeine is not per se linked with higher fat oxidation within the working muscle, as the potential higher release of FFA induced by caffeine (i.e., lipolytic effect) must be accompanied by a higher reliance on fat in the mitochondria of the working muscle.

Hypothetically, the effect of caffeine on fat oxidation during exercise may be reduced or withdrawn during fed-state exercise. First, exercise in the fed state with meals containing at least 25 g of carbohydrates produces a lower rate of fat oxidation with respect to the same exercise in the fasted state [17], suggesting that the fed state per se reduces fat oxidation during exercise. Second, caffeine may interact with some of the components of the meal (mainly carbohydrates), increasing postprandial glycaemic and insulinemic responses. In rats, the combination of a meal with caffeine intake induced an increase in postprandial glucose and insulin concentrations with respect to the isolated intake of the meal [18], which is similar to the results found in humans [19,20]. As insulin is a potent inhibitor of lipolysis [21,22], the combination of caffeine with a meal in the hours before exercise may reduce the potential effect of this substance on fat oxidation during exercise. Last, the co-ingestion of caffeine and carbohydrates increases exogenous carbohydrate oxidation [23] and dampens pain perception during exercise [24], which may shift the substrate used during exercise towards a greater reliance on carbohydrates [25]. Collectively, all this evidence sets a physiological basis to support that the benefits of caffeine on fat oxidation during exercise may be diminished during fed-state exercise. This is likely why most recent investigations on fat oxidation and caffeine during exercise set a fasting overnight before the exercise protocol [26,27,28] and may be linked to why no change in substrate oxidation during submaximal exercise after the intake of caffeine has been previously found in some investigations [11,29]. Although this would advocate for fasting > 5 h before exercise when using caffeine to enhance fat oxidation, most dietary sources of caffeine are habitually consumed in circumstances where food may be present and fed-state exercise is the condition under which athletes habitually compete and train [30]. Given the current literature gap about meta-analytical data on the effect of caffeine on fat oxidation during exercise in the fed state, the aim of the present study was to systematically review and meta-analyse the effect of caffeine on fat oxidation during aerobic exercise interventions performed in the fed state (<5 h since the last meal). The initial hypothesis was that caffeine would not provide a statistically significant benefit to fat oxidation during aerobic exercise in the fed state.

## 2. Materials and Methods

### 2.1. Search Strategy and Selection of Studies

This study was based on the Preferred Reporting Items for Systematic Review and Meta-Analyses (PRISMA) guidelines [31], including its last update [32]. The search for published studies on the topic was conducted in the databases PubMed (MEDLINE) and Web of Sciences (including KCI-Korean Journal Database, MEDLINE, Russian Science Citation Index, and SciELO Citation Index) up to the 1st of June 2023. Search terms included the following syntax: concept 1 (“caffeine” or “coffee” or “energy drink”) AND concept 2 (“free fatty acids” OR “fat oxidation” OR “lipid oxidation” OR “substrate oxidation” OR “respiratory exchange ratio”) AND concept 3 (“exercise”). The search was conducted without any restriction for the year of publication and the language filter was used to select only studies written in English. All titles and abstracts from the search were downloaded to a Microsoft Excel spreadsheet and manual cross-referencing was performed to identify duplicates. Titles and abstracts were then screened for a subsequent full-text review. Secondary searches consisted of screening the reference lists of the included studies. The search for published studies was independently performed by three authors (J.F.-S., A.R.-C., and D.T.-C.) and disagreements were resolved through discussion with the rest of the investigators.

For the articles obtained in the search, the following inclusion criteria were applied to select studies: (1) testing the effects of an acute dose of caffeine on fat oxidation, oxygen consumption (VO_2_), carbon dioxide production (VCO_2_), and respiratory exchange ratio (RER) during continuous or incremental exercise of aerobic submaximal intensity; (2) developing crossover experimental designs comparing pre-exercise caffeine ingestion with a pre-exercise ingestion of a placebo; (3) performing the placebo and caffeine conditions on the same participants (so each subject performed both conditions and acted as their own control) and under same circumstances regarding diet and exercise in the prior days; (4) including clear information about caffeine administration (relative or absolute dose), timing of caffeine intake, etc.; (5) where participants carried out a previous caffeine wash-out period of at least 12 h; (6) with an exercise protocol clearly defined in terms of duration and intensity; (7) including exercise intensity protocols identical in the experimental trials with caffeine and placebo; (8) where participants were in the fed state by having any type of meal in the 5 h before the exercise test. This period between the meal and the onset of the exercise protocol was established based on the meta-analysis by Collado-Mateo et al. [15] as these authors completed a similar meta-analysis but included only studies with a fasting protocol of >5 h. Hence, the current meta-analysis complements this prior meta-analysis and shows the full picture of the potential effect of caffeine on fat oxidation. (9) Written in English. The following exclusion criteria were applied: (1) studies using caffeine doses below 2 mg/kg. We used this threshold as there is evidence indicating that doses >2 mg/kg induce lower ergogenic effects [5,33]. Although there is no evidence to certify that doses <2 mg/kg limit the effect of caffeine on fat oxidation during exercise, we have used information for studies testing the dose–effect of caffeine on ergogenic responses during exercise. In addition, (2) the consumption of caffeine was combined with another ergogenic supplement (e.g., creatine); (3) ingestion of foods or caffeine during exercise; (4) exercise intensity (in terms of watts or different %VO_2max_) different between placebo and caffeine trials; (5) the protocol did not specify if the testing was carried out in the fed state or fasted state; (6) the study was conducted in a special environment such as hot environments or hypoxia; (7) RER value above 1 (does not allow calculation of fat oxidation); (8) studies including participants with a previous medical condition or disease; (9) investigations were performed with under 18-year-old participants; (10) animal studies; and (11) meta-analyses, systematic reviews, symposium, books, etc.

### 2.2. Data Extraction

Information about participants, interventions, comparisons, outcomes, and study design (PICOS; Table 1) was collected following the PRISMA methodology [31,32]. Data relating to study design, participants’ characteristics, specifications (if any) of the meal ingested before exercise to produce a fed state, sample size, caffeine dose, and exercise protocol were extracted from each study. Data on fat oxidation rate during exercise were extracted from the placebo and caffeine trials (mean and standard deviation (SD)). For those studies that only reported the standard error of the mean for placebo and caffeine data, SDs were calculated by multiplying the standard error by the square root of the number of subjects [34]. In studies presenting results in a graph format, images were zoomed to improve the precision of data estimates and extracted using WebPlotdigitizer [35]. In research not reporting data on fat oxidation rate, these data were calculated, when possible, from RER and VO_2_ or VCO_2_ data [36] or the corresponding author was contacted to obtain such information. In those investigations where the exercise was performed at a fixed/continuous submaximal exercise intensity with several measurements, the mean of all the fat oxidation measurements during exercise was calculated. In the investigations that use an exercise protocol at a fixed intensity to exhaustion, only data taken at the same time points in the placebo and caffeine conditions were used. In this regard, data on fat oxidation measured at exhaustion were excluded because this moment may not be the same in placebo and caffeine conditions. In the investigations with incremental exercise protocols (either ramp or steps), only data on peak fat oxidation rate during the whole exercise trial were obtained for caffeine and placebo conditions. In those studies that presented absolute values for caffeine doses (i.e., not normalised by participants’ body weight), the mean body weight value obtained from the sample descriptives was used to quantify the relative caffeine dose. Relative doses were used to establish s of analysis into <6 mg/kg and ≥6 mg/kg of caffeine, based on doses commonly used in dose–effect studies [27,37]. Data extraction was separately obtained by three authors (J.F.-S., A.R.-C., and D.T.-C.) and discrepancies were resolved through consultation with the remaining authors. In the case of discrepancies even after consultation, the authors voted to reach a final outcome.

### 2.3. Data Extraction

The quality of each experiment included in the meta-analysis was assessed using the Physiotherapy Evidence Database Scale (PEDro), which assigned a score from 0 to 10 points for each study [38]. The PEDro scale assesses the internal validity of studies according to factors such as randomisation, blinding, and presentation of results. All assessments for the application of the PEDro scale were conducted by J.F.-S. and A.R.-C. and verified by D.T.-C. Any disagreements were resolved by consensus and vote of all authors of the study.

**Table 1:** PICOS criteria for inclusion of crossover experimental trials in which caffeine was compared to a placebo in a single or double-blind fashion. For inclusion, study results must relate to the rate of fat oxidation measured during exercise.

Parameters	Inclusion	Exclusion	Extraction
P	Healthy active or trained adults	People with illnesses or children < 18 years of age	Number, age, sex, aerobic fitness level, caffeine habituation
I	Caffeine	Caffeine in combination with other ergogenic aids or multi-ingredient supplements	Dosage, form of administration, and timing
C	Placebo	Trials without a true placebo/control condition	Dosage, form of administration, and timing
O	Aerobic continuous or incremental exercise at submaximal intensity	Anaerobic or strength exercise, different intensities between conditions, special environment conditions (environmental, hypoxia, etc.)	Fat oxidation rate, oxygen consumption (VO_2_) or carbon dioxide production (VCO_2_), and respiratory exchange ratio (RER)
S	Single or double-blind crossover designs	Cross-sectional studies and meta-analyses, systematic reviews, symposiums, books, etc.	Type of experimental design and year of publication

### 2.4. Statistical Analyses

Descriptive data for participants’ characteristics are presented as mean ± SD. A global analysis was performed on all articles to determine the meta-analytic effects of caffeine on fat oxidation in the fed state (<5 h from the last meal to the onset of exercise). A meta-analysis software (Review Manager Software, version 5.4, USA) was used for statistical analysis using the inverse variance method with random effects and a 95% confidence interval (CI) [39]. The standardised mean difference (SMD) estimated by Hedges’ *g* was selected because of the heterogeneity of the testing protocols used in the included investigations, which could affect the variability in outcome measurements observed in each study. For each outcome, the SMD was calculated using mean and SD values from the placebo and caffeine trials, the sample size from each study, and the correlations between the trials. Given that none of the studies reported correlation values for the measurements of fat oxidation between the placebo and caffeine conditions, a 0.50 correlation was assumed for all trials per recommendations by Follmann et al. [40]. This correlation value is cautious as all studies were cross-over experiments with identical exercise conditions and methods of measurement for the placebo and caffeine conditions that would likely imply correlations > 0.50 between trials; however, the use of a 0.50 value reduces the partiality of the analysis and it may be considered as the least biased estimate of the true population correlation. For each outcome, a minimum of three studies was required to perform the meta-analysis [41]. In studies with several caffeine–placebo comparisons, such as different doses of forms of caffeine administration, all comparisons that met the inclusion and exclusion criteria were used. In the case that the caffeine and placebo conditions were not matched (e.g., two different doses of caffeine versus a single placebo condition), the placebo sample size was divided by the number of caffeine trials to avoid duplicating the value of the results in the quantitative analysis. The *p*-value fixed to consider the result statistically significant was <0.05. SMD effect size was interpreted as small with results < 0.4, moderate 0.4 to 0.7, and large > 0.75. A standardised measure of homogeneity (I^2^ statistic) was calculated to assess the level of heterogeneity in the included studies. An I^2^ value between 25% and 50% represented a small amount of inconsistency, while an I^2^ value between 50% and 75% represented a medium amount of heterogeneity and an I^2^ value > 75% represented a large amount of heterogeneity [35].

A sub-analysis was conducted according to the caffeine dose used in each experiment to further investigate the existence of a dose-dependent effect of caffeine on fat oxidation during fed-state exercise [37]. In addition, additional sub-analyses were conducted according to participants’ level of aerobic fitness (untrained/active with VO_2max_ < 50 mL/kg/min vs. trained individuals with VO_2max_ ≥ 50 mL/kg/min), daily intake of caffeine (i.e., habituation; naïve vs. habitual caffeine consumers), and timing of the meal respect to the onset of exercise. We used these criteria for the different sub-analyses as previous investigations have found that the response to caffeine may depend on the dose administered, participants’ fitness level, habituation to caffeine because of participants’ daily intake, and the interaction of caffeine with food components, mainly with carbohydrates [42,43,44,45].

## 3. Results

### 3.1. Study Selection

**Figure 1:**
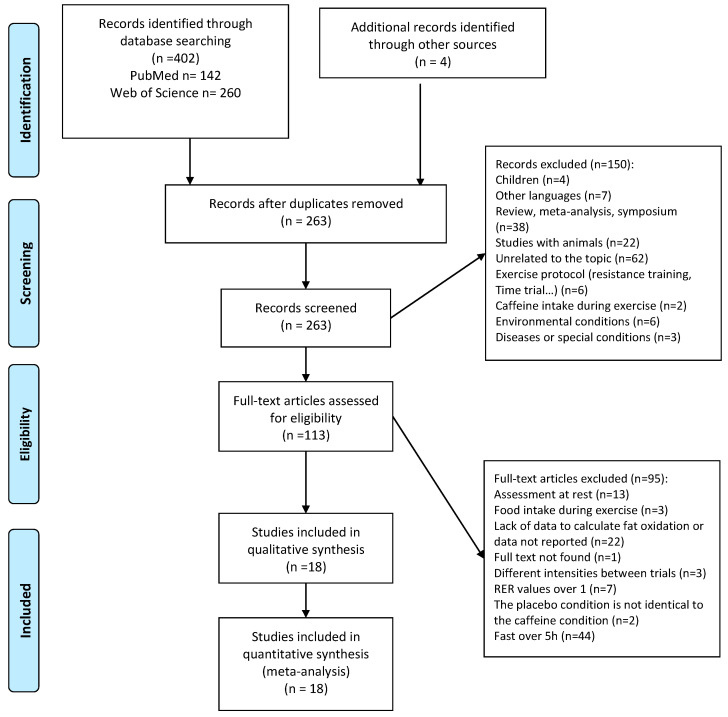
Study selection.

A total of 402 articles were obtained in the initial searches and 4 additional articles were found by checking the reference lists of the most relevant articles. After removing duplicates, 263 studies were selected for title and abstract review. By reading the title and abstracts, 150 studies were subsequently excluded because they (a) included children as the study sample (n = 4); (b) the exercise protocol was not appropriate for the meta-analysis because they used resistance exercise or time trials with free-chosen exercise intensity (n = 6); (c) were not written in English (n = 7); (d) included animals as a sample of the study (n = 22); (e) did not assess fat oxidation rate in an exercise context (n = 62); (f) the intake of caffeine was during exercise (n = 2); (g) were carried out under special environmental conditions like cold or hot environments (n = 6); (h) included participants with chronic conditions or diseases (n = 3); or (i) were review articles, meta-analyses, and conferences (n = 38). A total of 113 full-text articles were assessed for eligibility by application of inclusion/exclusion criteria and 95 studies were excluded for different reasons: (a) the assessment of fat oxidation rate was before or after exercise, with no measurement during exercise (n = 13); (b) participants ingested food or drinks during exercise (n = 3); (c) there were not enough data to calculate fat oxidation rate during exercise (e.g., only RER value was reported; n = 22); (d) the exercise intensity did not match between caffeine and placebo conditions (n = 3); (e) there was an RER value above 1.0 (n = 7); (f) the full text was not found (n = 1); (g) the placebo condition was not identical to the caffeine condition (n = 2); or (h) participants were fed more than 5 h before exercise (i.e., they were in a fasted state) (n = 44). After this process, only 18 studies published between 1982 and 2021 were included in the qualitative and quantitative analysis. A summary of this process is presented in Figure 1. 

### 3.2. Risk of Bias

PEDro scale scores for included studies are shown in Table 2. The mean score of the 18 studies was 8.28 with an SD of 0.83 points. All studies were within the range of 6 to 10 points and their quality ranged from fair to high. All articles met the statistical criteria (items 10 and 11), indicating that all studies provided unbiased estimates of the size of treatment effects in the comparison placebo–caffeine. Twelve articles specified the eligibility criteria of their participants (item 1). All included articles followed a crossover design in which the same group consumed a placebo and caffeine. The order of placebo or caffeine consumption was randomised in 13 out of 18 articles (item 2). In terms of the blinding process, 14 studies followed a double-blind design, in which both the participant and the researchers were blinded to the substances under investigation. Four of the studies used a single-blind design, in which only participants were blinded, and there was also one triple-blind study, in which participants, researchers, and evaluators were blinded.

### 3.3. Characteristics of the Participants

The overall data of the participants included in the studies of this systematic review and meta-analysis are depicted in Table 3. The total sample consisted of 228 individuals (185 males and 43 females). The included investigations involved aerobically trained athletes [46,49,51,52,53,54,56,59,61,62] and physically active individuals [47,48,50,55,57,58], although there was one investigation that used both aerobically trained and untrained individuals [63]. Participants were cyclists [46,52], runners [53,59], recreational athletes or sports players [47,55,57], and rugby players [61]. Seven of the studies included participants who were naïve caffeine consumers [47,48,54,56,57,60,61], while three studies included habitual caffeine users [51,52,58]. Of the remaining studies, three of them combined both habitual and naïve participants [53,59,63]. Still, five studies did not report any information regarding participants’ habituation to caffeine [46,49,50,55,62].

### 3.4. Description of the Interventions

Table 4 shows the main characteristics of the interventions. In all included studies, participants performed the exercise protocol in the fed state (less than 5 h between the last meal and the onset of exercise). The time since the last meal to the exercise onset was ≤2 h in eight articles [46,47,48,50,51,52,56,61], and >2 h in six articles [49,54,58,60,62,63]. In four articles, participants were asked to have a meal within the 5 h before exercise but no more information was provided about the feeding protocol [53,55,57,59]. In terms of exercise testing, 12 studies followed a continuous exercise intensity protocol [46,47,48,49,50,53,56,58,59,61,62,63] and 6 used an incremental exercise intensity protocol [51,52,54,55,57,60]. In addition, 14 articles performed the test on a cycle ergometer [46,48,50,51,52,54,56,57,58,59,60,61,62,63] and 4 on a treadmill [47,49,53,55].

**Table 3:** Summary design and participants of the included studies.

Study/Item	Design	Age	Sample Size	Physical Activity Level	Habitual Caffeine Intake
Acker-Hewitt et al., 2012 [46]	sRDB	28 ± 9	12 males	Trained cyclists VO_2max_ 66 ± 9 mL/kg/min	Not reported
Ahrens et al., 2007 [47]	RDB	19 ± 28	20 females	Enrolled in a personal fitness aerobics class	<50 mg/day
Black et al., 2015 [48]	RDB	22 ± 2	14 males (5) and females (9)	VO_2max_ < 50 mL/kg/min	<40 mg/day
Casal & Leon, 1985 [49]	RDB	30 ± 1	9 females	VO_2max_ 61.1 ± 1.3 mL/kg/min	Not reported
Demura et al., 2007 [50]	RB	21 ± 1	10 males	VO_2max_ 43.8 ± 9.5 mL/kg/min	Not reported
Glaister et al., 2016 [51]	RSB	38 ± 8	16 males	Endurance trained VO_2max_ > 50 mL/kg/min	225 mg/day
Glaister et al., 2021 [52]	RDB	42 ± 9	40 males	Cyclists VO_2max_ > 50 mL/kg/min	Habitual consumers
Graham and Spriet, 1991 [53]	RDB	28 ± 6	7 males (6) and females (1)	Runners VO_2max_ 76.0 ± 1.5 mL/kg/min	Consumers and non-consumers
Gutiérrez-Hellín and del Coso, 2018 [54]	RDB	25 ± 7	13 males (11) and females (2)	Active participants VO_2max_ > 50 mL/kg/min	<50 mg/day
Hulton et al., 2020 [55]	DB	20 ± 1	8 males	Recreational soccer players	Not reported
Jacobson et al., 2001 [56]	RDB	21 ± 4	8 males	VO_2max_ 65.2 ± 3.2 mL/kg/min	Non-habitual
Mcclaran and Wetter, 2007 [57]	RDB	19 ± 25	9 males	Aerobically active VO_2max_ 49.7 ± 6.3 mL/kg/min	Non-habitual caffeine use
Nishijima et al., 2002 [58]	RDB	25 ± 1	8 males	Physically active	Habitual consumers <500 mg/day
Oskarsson and McGawley, 2018 [59]	DB	males (30 ± 6); females (31 ± 9)	9 males (7) and females (2)	Runners VO_2max_ > 50 mL/kg/min	Consumers and non-consumers
Ramírez-Maldonado et al., 2021 [60]	RTB	32 ± 7	15 males	Experience in endurance training	<50 mg/day
Ryu et al., 2001 [61]		19 ± 1	5 males	Rugby players VO_2max_ 53.2 mL/kg/min	Non-habitual
Santos De Oliveira Cruz et al., 2015 [62]	RDB	25 ± 4	8 males	Active VO_2max_ 51 ± 5 mL/kg/min	Not reported
Toner et al., 1982 [63]	DB	28 ± 4	8 males	Trained and untrained	Consumers and non-consumers

DB: double blind; RDB: randomized double blind; sRDB: semi-randomized double blind; RSB: randomized single blind; RTB: randomized triple blind; VO_2max_: maximum oxygen uptake.

Caffeine was administered in capsules in 12 studies [46,47,48,51,52,53,54,56,57,58,59,62] and in powder form dissolved in fluids in 6 studies [49,50,55,60,61,63]. Caffeine was administered between 30 and 120 min before the onset of exercise, although a 60 min ingestion before exercise was the most common timing for caffeine administration. The dose of caffeine administered was quantified according to the participant’s body mass in most studies, ranging from 3 to 9 mg of caffeine per kilogram. In the remaining studies, an absolute dose of 400 mg [49], 350 mg [63], or 300 mg [58] was administered. In these studies, the mean of the body weight was used to quantify the dose of caffeine as mentioned in the methods section. Hence, from the total 18 studies, there were 6 studies with a caffeine dose of 6 or more mg/kg (≥6 mg/kg) [46,49,50,53,56,62]; in 11 studies, the dose of caffeine was less than 6 mg/kg (<6 mg/kg) [48,51,52,54,55,57,58,59,60,61,63]. One study used two different doses of caffeine, corresponding to 3 and 6 mg/kg [47], and data from this study were used separately for both s, respectively.

**Table 4:** Study characteristics.

Study	Intake	Form	Dose	Protocol	From of Exercise	Type of Exercise	Time since the Last Meal
Acker-Hewitt et al., 2012 [46]	60 min before	Capsule	6 mg/kg	20 min at 60%Wmax	CYC	CONT	2 h
Ahrens et al., 2007 [47]	60 min before	Capsule	3 and 6 mg/kg	8 min SS at 3.5 mph	TRE	CONT	1–2 h
Black et al., 2015 [48]	60 min before	Capsule	5 mg/kg	30 min at 60%VO_2max_	CYC	CONT	2 h
Casal and Leon, 1985 [49]	60 min before	Powder	400 mg	45 min at 75%VO_2max_	TRE	CONT	4 h
Demura et al., 2007 [50]	60 min before	Drink	6 mg/kg	1 h at 60% VO_2max_	CYC	CONT	2 h
Glaister et al., 2016 [51]	45 min before	Capsule	5 mg/kg	A six-stage test until OBLA	CYC	INC	1 h
Glaister et al., 2021 [52]	60 min before	Capsule	5 mg/kg	40, 55, 70 and 85% VO_2max_	CYC	INC	1 h
Graham and Spriet, 1991 [53]	60 min before	Capsule	9 mg/kg	85% VO_2max_	TRE	CONT	No fasted
Gutiérrez-Hellín and del Coso, 2018 [54]	60 min before	Capsule	3 mg/kg	30–90% VO_2max_	CYC	INC	4 h
Hulton et al., 2020 [55]	45 min before	Powder	5 mg/kg	Yo-yo intermittent endurance test	TRE	INC	No fasted
Jacobson et al., 2001 [56]	60 min before	Capsule	6 mg/kg	120 min at 70% VO_2max_	CYC	CONT	1 h
Mcclaran and Wetter, 2007 [57]	30 min before	Capsule	3 mg/kg	60 W, 120 W and 180 W	CYC	INC	No fasted
Nishijima et al., 2002 [58]	120 min before	Capsule	300 mg	30 min SS at 40–50% VT	CYC	CONT	125 min
Oskarsson and McGawley, 2018 [59]	45 min before	Capsule	4.3–5.6 mg/kg	5-min at 70% and 80% VO_2max_)	CYC	CONT	No fasted
Ramírez-Maldonado et al., 2021 [60]	30 min before	Powder	3 mg/kg	25 W increment until RER ≥ 1	CYC	INC	3 h
Ryu et al., 2001 [61]	60 min before	Powder	5 mg/kg	45 min at 60% VO_2max_	CYC	CONT	2 h
Santos De Oliveira Cruz et al., 2015 [62]	60 min before	Capsule	6 mg/kg	Until exhaustion at MLSS	CYC	CONT	3–4 h
Toner et al., 1982 [63]	60 min before	Powder	350 mg	40 and 70% VO_2max_	CYC	CONT	3 h

CONT: continuous; CYC: cycle ergometer; GXT: graded exercise testing; INC: incremental; TRE: treadmill; VO_2max_: maximum oxygen uptake; Wmax: maximal wattage obtained during a GXT; W: wattage; MLSS: maximal lactate steady state; OBLA: onset of blood lactate accumulation; SS: steady-state exercise; VT: ventilatory threshold.

### 3.5. Overall Effect of Caffeine on Fat Oxidation Rate during Fed-State Exercise

**Figure 2:**
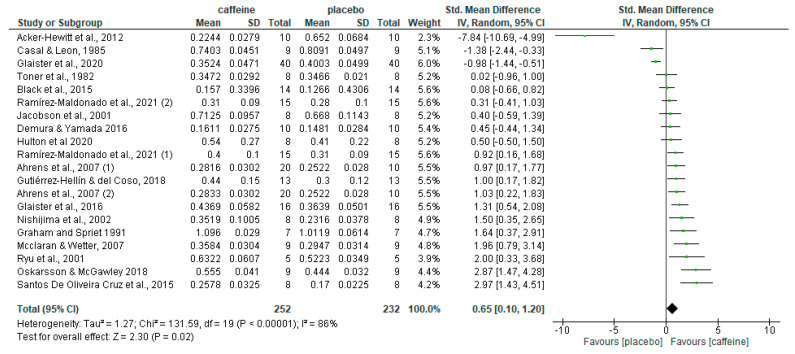
Effect of caffeine ingestion as compared to placebo on fat oxidation rate during fed-state exercise. The forest plot shows standardised mean differences with 95% confidence intervals (CI) for 18 studies with 20 comparisons. The diamond at the bottom of the graph represents the pooled standardised mean difference with 95% CI for all trials following random effects meta-analyses. The size of the plotted squares reflects the relative statistical weight for each study. (1) first placebo-caffeine comparison within the same study. (2) second placebo-caffeine comparison within the same study.

A total of 20 caffeine–placebo comparisons on fat oxidation rates during fed-state exercise were obtained from the 18 articles included in this systematic review. This is because two articles included two comparisons, one study comparing two different doses of caffeine to the placebo condition [47] and one study comparing caffeine and placebo at two different times of the day [60]. Figure 2 shows the overall effect of acute caffeine intake on fat oxidation rate during exercise in the fed state with these 20 placebo–caffeine comparisons. The pre-exercise intake of caffeine increased fat oxidation rate during exercise with respect to the placebo (*p* = 0.02), with an SMD of 0.65 and 95% CI from 0.10 to 1.20.

### 3.6. Dose-Related Effect of Caffeine on Fat Oxidation during Fed-State Exercise

**Figure 3:**
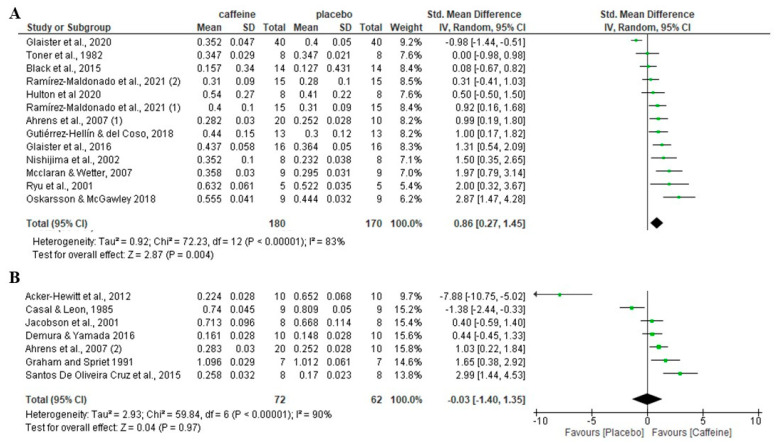
Effects of acute caffeine intake compared with placebo on the rate of fat oxidation during fed-state exercise in interventions with doses: (**A**) <6 mg/kg; (**B**) ≥6 mg/kg. The graph shows standardised mean differences with 95% confidence intervals (CI). The diamond at the bottom of the graph represents the pooled standardised mean difference with 95% CI for all trials after random-effects meta-analyses. The size of the squares reflects the relative statistical weight of each study. (1) first placebo-caffeine comparison within the same study. (2) second placebo-caffeine comparison within the same study.

Attending to the caffeine dose, a significant increase in fat oxidation rate during exercise was observed for doses < 6 mg/kg compared to placebo (*p* = 0.004), with an SMD of 0.86 and 95% CI of 0.27 to 1.45 (Figure 3). In contrast, there was no significant difference between the caffeine and the placebo on fat oxidation rate when the dose of caffeine was ≥6 mg/kg (*p* = 0.97), with an SMD of −0.03 and a 95% CI of −1.40 to 1.35.

### 3.7. Effect of Caffeine on Fat Oxidation during Fed-State Exercise Depending on Participants’ Fitness Level

**Figure 4:**
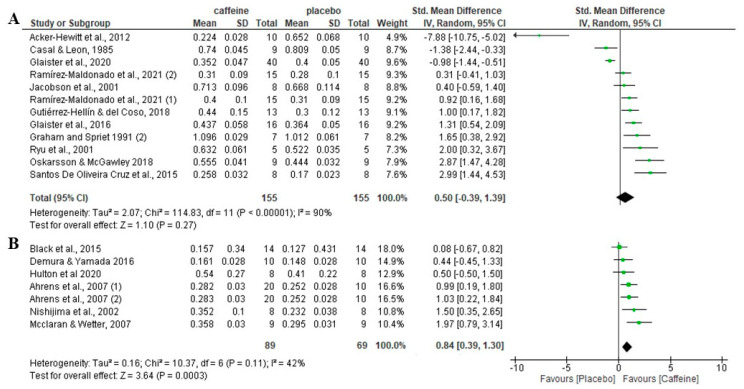
Effects of acute caffeine intake compared to placebo on the rate of fat oxidation during fed-state exercise in (**A**) aerobically trained individuals; (**B**) active/untrained. The graph shows standardised mean differences with 95% confidence intervals (CI). The diamond at the bottom of the graph represents the pooled standardised mean difference with 95% CI for all trials after random-effects meta-analyses. The size of the plotted squares reflects the relative statistical weight of each study. (1) first placebo-caffeine comparison within the same study. (2) second placebo-caffeine comparison within the same study.

In terms of participants’ aerobic fitness level, there was no significant difference between caffeine and placebo (*p* = 0.27) in aerobically trained participants (VO_2max_ ≥ 50 mL/kg/min)t, with an SMD of 0.50 and 95% CI of −0.39 to 1.39. In physically active/untrained individuals (VO_2max_ < 50 mL/kg/min), there was a significant increase in fat oxidation rate with caffeine compared to the placebo condition (*p* < 0.001), with an SMD of 0.84 and a 95% CI of 0.39 to 1.30 (Figure 4).

### 3.8. Effect of Caffeine on Fat Oxidation during Fed-State Exercise Depending on Participants’ Habituation to Caffeine

**Figure 5:**
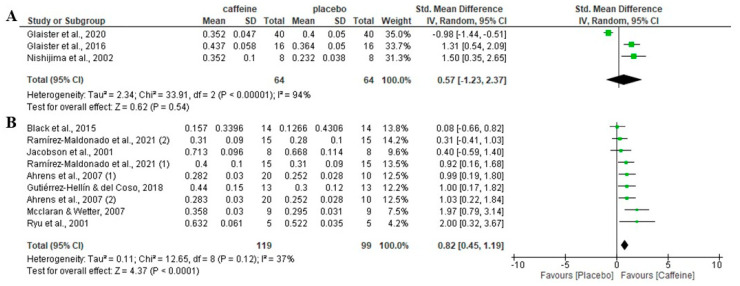
Effects of acute caffeine intake compared with placebo on the rate of fat oxidation during fed-state exercise: (**A**) in caffeine-habituated individuals; (**B**) in non-caffeine-habituated individuals. The graph shows standardised mean differences with 95% confidence intervals (CI). The diamond at the bottom of the graph represents the pooled standardised mean difference with 95% CI for all trials after random-effects meta-analyses. The size of the plotted squares reflects the relative statistical weight of each study. (1) first placebo-caffeine comparison within the same study. (2) second placebo-caffeine comparison within the same study.

Regarding the role of participants’ habituation of caffeine, there was no significant effect of caffeine on fat oxidation rate during fed-state exercise in participants habituated to caffeine (*p* = 0.54), with an SMD of 0.57 and 95% CI from −1.23 to 2.37. However, in participants non-habituated to caffeine intake, caffeine increased fat oxidation rate over the placebo (*p* < 0.001), with an SMD of 0.82 and 95% CI of 0.45 to 1.19 (Figure 5).

### 3.9. Effect of Caffeine on Fat Oxidation during Fed-State Exercise Depending on the Fasting Period Duration

**Figure 6:**
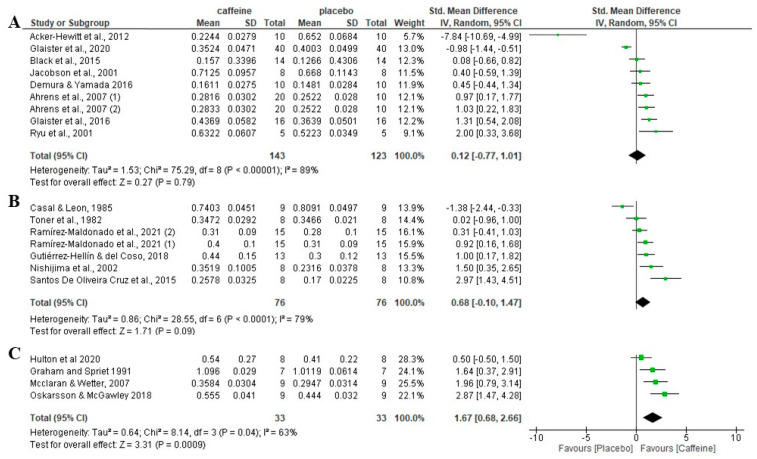
Effects of acute caffeine intake compared to placebo on the rate of fat oxidation during fed-state exercise when the time between the last meal and the onset of exercise was (**A**) ≤2 h; (**B**) >2 h; and (**C**) not specified. The graph shows standardised mean differences with 95% confidence intervals (CI). The diamond at the bottom of the graph represents the pooled standardised mean difference with 95% CI for all trials after random-effects meta-analyses. The size of the plotted squares reflects the relative statistical weight of each study. (1) first placebo-caffeine comparison within the same study. (2) second placebo-caffeine comparison within the same study.

In studies where feeding time between the meal and the onset of exercise was ≤2 h, there was no significant difference between the caffeine placebo condition of fat oxidation rates (*p* = 0.79), with an SMD of 0.12 and 95% CI −0.77 to 1.01. When the feeding time between the meal and the onset of exercise was >2 h, there was also no significant differences between the caffeine condition and the placebo condition (*p* = 0.09), with an SMD of 0.68 and 95% CI of −0.10 to 1.47. In those in which the instruction was only to have a meal within the 5 h before exercise, without specific information about the number of hours from the meal to the onset of exercise, there was a significant effect of the caffeine condition on fat oxidation relative to the placebo condition (*p* < 0.001), with an SMD of 1.67 and 95% CI of 0.68 to 2.66 (Figure 6).

## 4. Discussion

The purpose of this systematic review and meta-analysis was to summarise studies on the effect of acute caffeine intake on fat oxidation during submaximal exercise in the fed state (<5 h between the last meal and the onset of exercise). The current meta-analysis complements the results from a previous meta-analysis by Collado-Mateo et al. [15] as these authors completed a similar meta-analysis but included studies with a fasting protocol of >5 h. The main finding of the current meta-analysis was that the fat oxidation rate during exercise was moderately increased following acute caffeine intake (Figure 2) even when participants were in a fed state (<5 h from the last meal to the onset of the exercise). Overall, caffeine consumption increased fat oxidation during fed-state exercise by 8.1%. However, the current meta-analysis also showed that there are several modulators of the effect of caffeine on fat oxidation such as caffeine dose relative to participants’ body mass, participants’ aerobic fitness level, and participants’ habituation to caffeine through daily intake of this substance. For example, the sub-analysis of the dose-related effect of caffeine on fat oxidation indicated that the effect of this substance occurred with doses between 2 and <6 mg/kg, while this effect disappeared, in statistically significant terms, when using doses ≥ 6 mg/kg. Furthermore, caffeine intake was associated with higher fat oxidation during fed-state exercise with participants with moderate-to-low levels of aerobic fitness (VO_2max_ < 50 mL/kg/min) whereas no such effect was found in studies carried out with samples of aerobically trained athletes (VO_2max_ ≥ 50 mL/kg/min). Likewise, caffeine intake was associated with higher fat oxidation during fed-state exercise in naïve non-caffeine consumers, while the effect was not present in participants habituated to caffeine through daily intake of the substance. Finally, the sub-analysis of the time from the last meal to the onset of the exercise reflected imperfect outcomes, as there was only a significant association between caffeine intake and increased fat oxidation in those articles reporting that participants were in a fed state without specific information about timing. Based on these results, we concluded that a moderate dose of caffeine ingested during exercise can enhance fat oxidation during submaximal aerobic exercise even when participants had a meal in the 5 h before exercise. In terms of magnitude, the effect reported in the current meta-analysis on fat oxidation with caffeine ingested before fed-state exercise (SMD = 0.65; 95% CI = 0.10 to 1.20) was comparable to the one reported by Collado-Mateo et al. [15] (SMD = 0.73; 95% CI = 0.19 to 1.27) with caffeine ingested before fed-state exercise. Although the studies of both meta-analyses were different, the comparable inclusion criteria used in terms of caffeine dose and type of exercise comparisons suggest that the effect of caffeine on fat oxidation during aerobic exercise was minimally affected by the presence/absence of a fasting period. This is a hypothesis that merits further investigation.

Previous research suggests that acute caffeine intake in combination with carbohydrate ingestion before exercise increases glycolytic metabolism to the detriment of fatty acid metabolism [17,25]. In fact, there are several mechanisms that support that the co-ingestion of caffeine and carbohydrate-containing meals increases the reliance on carbohydrates as a fuel during aerobic exercise, as caffeine increases postprandial glycaemic and insulinemic responses in rats [18] and humans [19,20] and increases exogenous carbohydrate oxidation [23]. However, there is also evidence supporting that caffeine prevents glycogen breakdown and it may act as a strong inhibitor of hepatic and skeletal muscle glycogen phosphorylase, the enzyme responsible for glycogenolysis [64]. Therefore, although there is physiological support to hypothesise that the effect of caffeine on fat oxidation during exercise may be reduced (or withdrawn) when a meal is ingested in the hours before exercise (with respect to the effect already found in exercise performed after fasting [15]), the current meta-analysis rejects this hypothesis. The effect of caffeine to increase fat metabolism irrespective of the presence of fasting is supported by another recent meta-analysis [16]. However, their results included resting conditions and the fat oxidation only represented one of several forms of action of caffeine on fat metabolism. Therefore, the current investigation sheds light on ascertaining that caffeine intake before exercise can enhance the rate of fat oxidation during exercise irrespective of the presence (or not) of a fasting period. Therefore, this study is the first to indicate that feeding before exercise may not counteract the effect of caffeine on fat oxidation during exercise.

Caffeine is rapidly absorbed after consumption and, due to its lipophilic nature, can cross the haematoencephalic membrane, generating changes in the central nervous system [65]. The ergogenic effect of caffeine has traditionally been understood to occur through its binding to adenosine A_1_ and A_2a_ receptors, which blocks the inhibitory action of adenosine on neurons [66]. This may lead to increased physical and cognitive performance as the inhibition of adenosine’s effect produce the release of several excitatory neurotransmitters in the brain particularly dopamine. However, this mechanism of action of caffeine as an ergogenic aid appears to be useful for “all-out” exercise situations but it may be different from the mechanism(s) affecting the rate of fat oxidation during submaximal aerobic exercise presented here. This would explain why the effect of caffeine on fat oxidation can only be seen at low-to-moderate exercise intensity and why the ergogenic effect of caffeine and its effect on fat oxidation are somewhat incompatible. In this regard, research has found increases in the release of epinephrine and norepinephrine following caffeine ingestion, which may stimulate the liberation of fatty acids and lipolysis during exercise, explaining then the augment in the use of fat as a fuel during exercise after caffeine intake [67]. Caffeine also can increase oxygen saturation at the muscle level during submaximal exercise [68] and can reduce pain and fatigue perception, which may be an additive mechanisms to explain the higher reliance on fat during exercise [69]. Collectively, all this information suggests that the mechanisms for higher reliance in fat during submaximal exercise may be associated to “local” mechanism rather than the “central” mechanisms associated with the ergogenic effect of caffeine during maximal-intensity exercise. 

To understand the potential interactions of meal intake and caffeine effect on fat oxidation during exercise in more depth, a sub-group analysis was conducted based on the hours from the last meal to the onset of exercise. There was no significant effect of caffeine on fat oxidation in either those studies with ≤2 h between the meal and the onset of exercise or those with 2–5 h between the meal and the onset of exercise. However, a third sub-analysis was performed on those investigations that indicated that subjects were in a fed state but did not specify the time since the last meal was the only sub-analysis that showed a significant effect of caffeine on fat oxidation during fed-state exercise. This represents a limitation of the current meta-analytical approach, as we have been unable to specify what timing is better to combine feeding and caffeine intake to optimise the effect of caffeine on fat oxidation during exercise. In this case, caffeine is habitually ingested 60 min before exercise to obtain peak plasma concentration of caffeine before exercise [70] and the meal should be ingested before caffeine intake, approximately 4 h before the onset of exercise to follow traditional guidelines for carbohydrate intake before exercise [71]. Although this may be a current recommendation based on the available literature, future research should investigate what is the best time to combine pre-exercise feeding and caffeine ingestion to optimise fat oxidation during and after exercise.

Based on the level of participants’ aerobic fitness, associated with their relative VO_2max_, a sub-analysis indicated that caffeine significantly increased fat oxidation during exercise in active/untrained participants, whereas, in more trained subjects, the effect was not significant. In this regard, Collado-Mateo et al. [15] reported similar results to the present meta-analysis about the effect that the training level of the participants had on the modulation of the response to caffeine intake. The difference in the magnitude of the effect of caffeine based on participants’ training level has already been studied in other exercise modalities. In the context of strength training, caffeine appears to exert its ergogenic effect in untrained individuals, whereas this effect is of lower magnitude in those with a higher level of training [42]. This could be because untrained individuals have a greater window of improvement to achieve performance, whereas trained subjects may have a greater degree of adaptation and lower margin for improvement through nutritional interventions, limiting the potential of caffeine to enhance fat oxidation. However, research can also be found reflecting an ergogenic effect of caffeine of similar magnitude and an effect on fat oxidation derived from caffeine intake in highly trained athletes and less-trained individuals [5,16]. Therefore, more research is needed to find out whether the effect of caffeine on the rate of fat oxidation is indeed dependent on the level of training or if this is just an artefact of the number of studies that used either type of participant.

The sub-analysis of the effect of caffeine on fat oxidation by the level of participants’ habituation to caffeine showed that, in individuals not habituated to caffeine intake, caffeine increased the rate of fat oxidation during fed-state exercise. However, in the caffeine-habituated participants, there was no significant difference between the placebo and caffeine conditions. The lack of effect of caffeine on fat oxidation during exercise in caffeine consumers may be because daily caffeine consumption may lead to tolerance to the physiological effects derived from caffeine during exercise. For example, previous investigations have shown that the ergogenic effect of caffeine to enhance peak wattage during a ramp exercise test and wattage at the second ventilatory threshold was progressively reduced when caffeine was ingested daily for 20 days [45,72]. Additionally, habitual caffeine consumption attenuates the epinephrine responses produced by the combination of exercise and caffeine [73], which may lead to a slight reduction in the release of free fatty acids induced by the administration of caffeine. Although the existence of tolerance to the effect of caffeine on fat oxidation during exercise must be tested using a cross-over experiment with a controlled daily caffeine intake, the data of the current meta-analysis suggest that the magnitude of the effect of caffeine to enhance the use of fat as a fuel during exercise is higher in naïve vs. habitual consumers. The sub-analysis of the effect of caffeine depending on the dose of caffeine ingested before exercise indicated that this substance only increased the rate of fat oxidation during fed-state exercise when the dose of caffeine was less than 6 mg/kg. In contrast, when the dose of caffeine ingested before exercise was greater than or equal to 6 mg/kg, there was no significant effect of caffeine on fat oxidation during fed-state exercise. These outcomes should be interpreted cautiously, as the dose–effect presented here has been calculated with data from different studies for the <6 mg/kg and ≥6 mg/kg. Additionally, although both sub-analyses have an appropriate statistical power with 13 and 7 caffeine–placebo comparisons, respectively, the sub-analysis with ≥6 mg/kg included the study by Acker-Hewitt et al., 2012 [46], in which the placebo produced a much greater effect on fat oxidation than caffeine (i.e., −7.84 SMD units; see Figure 3). Lastly, there may be also an interaction between the dose of caffeine administered and participants’ habituation to caffeine in the studies. For example, five out of the seven papers that used non-habituated-to-caffeine participants used also caffeine doses lower than 6 mg/kg [47,54,57,60,61].

Even with the concerns raised above about the interpretation of the dose–effect results of the current meta-analysis, there is physiological support for the lack of effect of caffeine during fed-state exercise when the dose of caffeine is high. First, it is well known that caffeine may exacerbate postprandial glycaemic and insulinemic responses [19], while this potentially “negative” effect of caffeine is present with meals of high and low glycaemic index [20]. Interestingly, Beaudoin et al. [44] found that caffeine ingestion increased the insulinemic response to a meal in a dose-dependent fashion beginning at very low doses. Therefore, it is possible that the intake of low-to-moderate doses of caffeine (i.e., <6 mg/kg) produce a smaller effect on increasing postprandial insulin concentration, while higher doses of caffeine (i.e., ≥6 mg/kg) exacerbate postprandial insulin release. Considering that insulin inhibits lipolysis and fat oxidation [21,22], this mechanism may explain why the higher doses of caffeine were ineffective in enhancing fat oxidation during fed-state exercise. Interestingly, higher doses of caffeine were better in enhancing fat oxidation during exercise in the fasting state [15], likely because, in this context, with the absence of food intake for >5 h before exercise, there is no interference of the meal components with the effect of caffeine. Although it is a hypothesis at this stage, the use of <6 mg/kg of caffeine may produce a better balance between the effect of caffeine on postprandial insulin concentration and the inhibition of hepatic and skeletal muscle glycogen phosphorylase [64], entailing an optimised situation to enhance fat oxidation during fed-state exercise. Although further investigations are warranted to definitively ascertain whether doses ≥6 mg/kg of caffeine limit the effect of this substance on fat oxidation during exercise in the fed state, our data suggest that using these high doses of caffeine may not be a good strategy to assure enhanced fat oxidation during fed-state exercise. The use of low-to-moderate doses of caffeine, up to 6 mg/kg, may be a more recommendable choice when using this substance as a potential “fat-burner” during exercise performed in a postprandial state.

The present systematic review and meta-analysis is not exempt from limitations, which should be discussed for a better understanding of the results. First, the description of the meal ingested (i.e., information about macronutrient proportions or food included in the meal) and the time when the meal was ingested was inadequate in most investigations included in this systematic review. Therefore, we have been unable to perform further analysis on the potential interference of the types of meals (e.g., high vs. low carbohydrate content) on the effect of caffeine on fat oxidation during exercise. Additionally, as not all articles specified the exact moment when the meal was consumed before exercise (some studies only indicated that participants were in a fed state [53,55,57,59]), we have been unable to determine the best approach to use a pre-exercise meal to optimise the effect of caffeine on fat oxidation during exercise, as previously discussed. Additionally, only one study of those included in this meta-analysis determined the effect of the *CYP1A2* and *ADORA2A* genetic variations, even though polymorphisms in these genes can modify the response to caffeine [5]. In addition, the number of female participants accounted for only 18% of the total participants, which prevented the analysis of the effect of participants’ sex on the primary endpoint and, in turn, implies the existence of sex bias on the outcomes of this systematic review. Also, the I^2^ statistic was greater than 75% for the caffeine and placebo comparison of fat oxidation rate during exercise, indicating a large heterogeneity in the studies that met the inclusion criteria. Control criteria were very exhaustive in attempting to attribute the increase in the rate of fat oxidation during exercise to acute caffeine intake. However, due to the different exercise characteristics used among the included investigations, it was not possible to perform a sub-analysis to determine whether exercise intensity modifies the magnitude of the effect of caffeine on fat oxidation. Therefore, another limitation of the study is that the large variability of exercise protocols used in the included articles does not allow us to know which exercise intensity is most effective in increasing the rate of fat oxidation in the fed state. On the methodological approach, most of the studies had high-quality indices, so the results of the present investigation are not influenced by the inclusion of studies with low methodological quality, which gives certain value to the conclusions of the study.

In summary, pre-exercise ingestion of a moderate dose of caffeine may be an effective strategy to increase the fat oxidation rate during prolonged and submaximal exercise in the fed state. Based on these results, no specific hours since the last meal have been established as “optimal” to improve the effect of caffeine on fat oxidation rate during exercise, although evidence is clear when indicating that caffeine should be consumed ~1 h before exercise to facilitate peak concentrations of caffeine during exercise [70]. Caffeine showed a statistically significant increase in the rate of fat oxidation during fed-state exercise in physically active individuals, while this effect was not significant in trained athletes. In addition, caffeine showed a significant increase in fat oxidation in those less habituated to caffeine, whereas this did not occur in studies that used samples of individuals with low-to-moderate levels of daily caffeine intake. At doses between 2 and 6 mg/kg, there was a significant effect of caffeine on increasing the fat oxidation rate, whereas there was no significant effect at doses higher than 6 mg/kg. The high quality of the investigations selected and the many eligibility criteria used for this systematic review and meta-analysis (investigations where the intensity was a free choice or where caffeine was combined with other supplements were excluded) suggest that caffeine may have the capacity to enhance fat oxidation during aerobic exercise in the fed state (meal within 5 h before exercise). Still, further investigation is also needed to establish the minimal effective dose of caffeine, the gender–caffeine interaction on fat oxidation during exercise, the most common drawbacks of caffeine supplementation in the context of submaximal aerobic exercise or the most effective exercise intensity for increasing the rate of fat oxidation in the fed state.

From a practical perspective, exercise practitioners can consider caffeine as a substance effective in enhancing fat oxidation during exercise in the fed (current systematic review) and fasted states (previous systematic review [15]). However, the exercise selected to optimise fat oxidation should be aerobic and of submaximal intensity to favour the use of fat as a fuel in the working muscle. In general, this effect of caffeine to enhance fat oxidation during exercise is useful for exercise enthusiasts seeking body fat reduction/body composition changes through exercise programs, as the implications of better fat oxidation during exercise may have very limited benefits for athletes in the context of high-performance exercise or during sports competition. In any case, the combination of caffeine intake and exercise should be prolonged in the time, as the effect found in the current systematic review is acute and renders only for a few grams of “extra” fat oxidised per hour of exercise. Finally, the benefits of caffeine to enhance fat oxidation should be assessed with respect to the potential side effects that this substance produces when it is ingested acutely and chronically [74]. To this regard, a dose of caffeine ~3 mg/kg 1 h before exercise may be the optimal choice to obtain the benefits of this substance during exercise while minimising the prevalence and magnitude of side effects. Further investigations are still needed to ascertain what type of meal and when it should be ingested to avoid any interactions of the food components on the potential effect of caffeine to augment fat oxidation during exercise.

## Figures and Tables

**Table 2:** PEDro scale scores for included studies.

Study/Item	1	2	3	4	5	6	7	8	9	10	11	Score
Acker-Hewitt et al., 2012 [46]	N	N	Y	Y	Y	Y	N	N	Y	Y	Y	7
Ahrens et al., 2007 [47]	N	Y	Y	Y	Y	Y	N	N	Y	Y	Y	8
Black et al., 2015 [48]	Y	Y	Y	Y	Y	Y	N	Y	Y	Y	Y	9
Casal and Leon, 1985 [49]	Y	Y	Y	Y	Y	Y	N	Y	Y	Y	Y	9
Demura et al., 2007 [50]	Y	Y	Y	Y	Y	N	N	Y	Y	Y	Y	8
Glaister et al., 2016 [51]	Y	Y	Y	Y	Y	N	N	Y	Y	Y	Y	8
Glaister et al., 2021 [52]	N	Y	Y	Y	Y	Y	N	N	Y	Y	Y	8
Graham and Spriet, 1991 [53]	Y	Y	Y	Y	Y	Y	N	Y	Y	Y	Y	9
Gutiérrez-Hellín and del Coso, 2018 [54]	Y	Y	Y	Y	Y	Y	N	Y	Y	Y	Y	9
Hulton et al., 2020 [55]	Y	N	Y	Y	Y	Y	N	Y	Y	Y	Y	8
Jacobson et al., 2001 [56]	Y	Y	Y	Y	Y	Y	N	Y	Y	Y	Y	9
Mcclaran and Wetter, 2007 [57]	Y	Y	Y	Y	Y	Y	N	Y	Y	Y	Y	9
Nishijima et al., 2002 [58]	Y	Y	Y	Y	Y	N	N	Y	Y	Y	Y	8
Oskarsson and McGawley, 2018 [59]	Y	N	Y	Y	Y	Y	N	Y	Y	Y	Y	8
Ramírez-Maldonado et al., 2021 [60]	Y	Y	N	Y	Y	Y	Y	Y	Y	Y	Y	9
Ryu et al., 2001 [61]	Y	N	N	Y	Y	N	N	Y	Y	Y	Y	6
Santos De Oliveira Cruz et al., 2015 [62]	Y	Y	Y	Y	Y	Y	N	Y	Y	Y	Y	9
Toner et al., 1982 [63]	N	N	Y	Y	Y	Y	N	Y	Y	Y	Y	8

Y = yes; N = no. Items: (1) eligibility criteria were specified; (2) subjects were randomly allocated to groups; (3) allocation was concealed; (4) the groups were similar at baseline; (5) there was blinding of all subjects; (6) there was blinding of all therapists; (7) there was blinding of all assessors; (8) measures of at least one key outcome were obtained from more than 85% of the subjects who were initially allocated to groups; (9) intention-to-treat analysis was performed on all subjects who received the treatment; (10) the results of between-group statistical comparisons are reported for at least one key outcome; (11) the study provides both point measures and measures of variability for at least one key outcome. Score: each satisfied item (except the first) contributes 1 point to the total score, yielding a PEDro scale score that can range from 0 to 10 points.
